# Quantity and Intensity, as Applied to Faradaic Electricity

**Published:** 1873-11

**Authors:** Plym. S. Hayes

**Affiliations:** No. 123 Calumet Ave.


ARTICLE II.—*Quantity and Intensity, as applied to Faradaic Electricity.* Read before the Chicago Society of Physicians and Surgeons, Sept. 22, 1873, by PLYM. S. HAYES, M.D.

It is a significant fact, that very few physicians understand the construction of the electrical instruments they use, and the principles that govern and modify the generation of electricity by the various kinds of apparatus. Knowing this to be the case, and that some essential principles have been overlooked in the manufacture of faradaic instruments, which have lessened their value as therapeutic agents, has induced us to place before the profession some of the overlooked principles. We hope by this means to be able, in a measure, to explain why different results have been obtained, by different physicians in similar cases, by the application of faradaic electricity.

A little more than forty years ago, Faraday discovered that instantaneous currents were generated in metallic conductors, either under the influence of similar conductors transmitting currents of electricity, or by the influence of powerful magnets. He even went so far as to use the magnetism of the earth—the greatest of all known magnets—to generate these currents, which he termed currents of induction, or induced currents. This form of electricity is commonly called faradaic (from the discoverer of it) or induced electricity.

After the discovery of these principles, instruments for the generation of faradaic electricity came rapidly into use, as therapeutic agents, on account of the amount of electricity generated, the ease with which it could be manipulated, and the small size of the instrument.

The simplest form of the instrument consists of two coils, or helices of insulated wire, the one within the other, but thoroughly insulated from each other; within the inner coil, and extending through it, is the core or electro-magnet, of soft iron wires. Connected with the inner or inducing coil is a galvanic cell; there being intercalated in the circuit a rheotome, or current breaker. The ends of the wire which forms the outer or inductive coil, terminate in the poles.

It might be well, in order to get a clearer idea of what follows, to give at least the essential physiological differences between frictional and galvanic electricity. We have chosen these two forms because they occupy the extremes of that molecular force, and consequently their physiological actions are more distinctly marked. So different in their various phenomena are these two forms, that for a time some scientists thought that they might be two different forces, as unlike as magnetism and electricity. Since the discovery of Faraday they have been proven to be the same force, for by modifying the faradaic apparatus, electricity may be obtained, which in one case is identical with frictional, in another with galvanic, and in a third seems to combine in one—although in a limited degree—the properties of both of these forms. All of the differences in the physical and physiological phenomena of frictional and galvanic electricity, are due to the difference in degree of the *quantity* and *intensity* that each possesses.

*Quantity*, as applied to electricity, is that property in virtue of which electricity can produce a given amount of chemical action in a unit of time; while *intensity* in electricity is that property in virtue of which electricity can overcome resistance, such, for instance, as is encountered in traversing a poorly conducting medium. To illustrate this, the quantity and intensity of electricity might be compared with quantity and intensity as applied to heat. For instance, the intensity of the heat found in the jet of the oxy-hydrogen blow-pipe is much greater than that found in a blast furnace; but the quantity of heat found in the latter is much greater than that of the oxyhydrogen jet.

From the foregoing definitions it is readily seen that the galvanic current is of great quantity and small intensity, as compared with frictional electricity, in which intensity predominates, and quantity exists only in a slight degree. For instance, Weber has calculated that if the amount of positive frictional electricity required to decompose a grain of water were accumulated on a cloud at a distance of 3,000 feet from the earth's surface, it would exert an attractive force upon the earth of upwards of 1,500 tons. Thus it is that these two forms differ from each other simply in degree and not in character.

A few of the most important differences in the physiological phenomena of these two varieties of electricity, are as follows:

| | |
|---|---|
| The galvanic current, when made to act upon the skin, produces a sense of heat, redness and inflammation; and even destruction of the skin and contiguous tissues may follow the application, if the current be powerful and the application be sufficiently prolonged. | If sparks from a common electrical machine be applied to the skin, a sense of prickling and pain is felt, and if the sparks are large, the skin is reddened and a papular eruption resembling the *lichen urticatus* is produced. |
| The effects of galvanic electricity on the muscles are to produce, at the instant of opening and closing the circuit, a contraction; and during the application of the current—if it is sufficiently strong—a tremulous muscular motion, and sensation. | Frictional electricity simply produces a muscular contraction at each discharge, provided the sparks be large. |
| Galvanic electricity affects prominently the nerve centres and nerves of special sense. With regard to the latter a sensation may be called forth which corresponds to a sensation peculiar to the special nerve, under the influence of the current; we can—so to speak—see, taste, smell and hear this form of electricity. Its action on the nerve centres is manifested by vertigo, syncope, nausea, and even convulsions. | Frictional electricity does not perceptibly affect the nerve centres and nerves of special sense (if possibly we exclude its action on the optic nerve), unless the shock be so severe as to endanger the life of the subject operated on. |

Galvanic electricity tends to chemically decompose the fluids and solids of the body, at the place of contact of the applied electrodes.

The chemical effects of frictional electricity on the human body are not perceptible.

From the intermediate position that faradaic electricity occupies, it can, under certain conditions, produce similar effects on the skin, muscles, nerve centres, and some of the nerves of special sense, that an interrupted galvanic current of medium quantity and intensity does; and will also produce on the skin and muscles effects similar to that produced by frictional electricity. Combining, as it does, the properties of these extremes, its effects on the animal economy are not as distinctly marked as in the employment of the galvanic or frictional forms alone; and the results obtained in certain instances by faradaic electricity are different from those obtained by either of the other varieties.

Faradaic currents of different quantity and intensity may be obtained, first, by modifying the quantity and intensity of the galvanic or inducing current, and, second, by varying the length and diameter of the wire used in the construction of the coils.

In regard to the galvanic current, it should be generated from a single cell, or series of cells arranged as a single one, with large metallic surface. The amount of surface would have to be varied according to the form of battery used, to get the same amount of quantity in each case; for the different batteries differ from each other in electro-motive force. In this case *quantity* is the essential element to be sought for, if we desire a faradaic current of *great quantity;* for it is a self-evident truth, that from a given amount of a force you cannot generate a greater. Neither can you, from a given quantity of galvanic electricity, by means of a faradaic apparatus, generate electricity of greater quantity than that possessed by the inducing current, for then would perpetual motion be achieved. To illustrate this principle, let us represent the quantity of the galvanic current by ten, the quantity of electricity induced by this current in a faradaic apparatus would be represented by three or four.

The intensity of the induced current is proportional to the intensity of the inducing current.

The quantity and intensity of the faradaic current is still further modified by the length and diameter of the wire employed in the manufacture of the coils. It is a well known fact, and one which is the underlying principle in the manufacture of several medical and telegraphic instruments, that a current of electricity is resisted, in its passage through a conductor, directly in proportion to its length, and inversely as to its diameter; thus a long or fine wire resists the passage of a current much more than does a short or thick wire of the same metal. The difference in the conductivity of metals need not be dwelt on in this connection, as copper is the one usually employed in the construction of the coils. If the wire is long and fine, considerable intensity of current is required to overcome the resistance offered by it; and, conversely, a current generated in such a wire will be of considerable intensity; such, for instance, as is generated by a Ruhmkorff's coil; the length of the wire in the outer coil in some of the largest sizes of these machines is sixty miles, and the diameter of the wire one-fifth of a millimeter. From such an instrument sparks of a number of inches in length may be obtained.

By shortening and enlarging the wire, keeping up all of the time the proportion between the outer and inner coils, currents are obtained which are of greater quantity and less intensity than are found in the Ruhmkorff instruments, or those usually employed for medical use, provided the galvanic current remains the same.

To recapitulate that which has been said in regard to the wire: "The thinner the wire the greater the tension of the induced electricity," is the statement made by Ganot. Wm. Crookes, in the *Quarterly Journal of Science*, speaking of the induction of electricity from magnets—for the laws discovered by Faraday regarding the generation of induced from galvanic currents are, in a measure, as applicable to the generation of currents from magnets—says, "the quantity of the current likewise differs according to the kind of wire surrounding the armature, a *short thick wire producing effects of quantity, and a long thin wire, of intensity.*"

The faradaic instruments for therapeutic purposes in general use seem to have been manufactured solely to produce a given amount of intensity, overlooking entirely the element of quantity.

That in many cases great benefit has been derived from the ordinary instruments, none will deny; but that the same amount of good may be obtained with less pain to the patient, and that results which have not been achieved by faradaic currents of intensity have been by those of quantity, are clearly demonstrated by our experience.

With faradaic quantity currents, the deep seated muscles and those remote from the points of contact of the electrodes, but in the course of the current, may be felt to contract very markedly, while the intensity is not sufficient to produce the sensation of pain, so frequently experienced in the intensity currents. In fact, faradaic currents of great quantity affect the nerves of sensation only slightly.

That faradaic electricity will ever take the place of the galvanic we do not expect, for the latter has properties which it would be impossible to obtain in the former; yet we know that results can be obtained in the employment of electricity which have not been hitherto fully appreciated.

No. 123 Calumet Ave.

